# Iodine Deficiency and Iodine Prophylaxis: An Overview and Update

**DOI:** 10.3390/nu15041004

**Published:** 2023-02-16

**Authors:** Giuseppe Lisco, Anna De Tullio, Domenico Triggiani, Roberta Zupo, Vito Angelo Giagulli, Giovanni De Pergola, Giuseppina Piazzolla, Edoardo Guastamacchia, Carlo Sabbà, Vincenzo Triggiani

**Affiliations:** 1 Interdisciplinary Department of Medicine, Section of Internal Medicine, Geriatrics, Endocrinology and Rare Diseases, School of Medicine, University of Bari “Aldo Moro”, Piazza Giulio Cesare 11, 70124 Bari, Italy; 2 Unit of Data Sciences and Technology Innovation for Population Health, National Institute of Gastroenterology, Saverio de Bellis, Research Hospital, 70013 Bari, Italy; 3 Unit of Geriatrics and Internal Medicine, National Institute of Gastroenterology, Saverio de Bellis, Research Hospital, 70013 Bari, Italy

**Keywords:** iodine, iodine deficiency, iodized salt, iodine prophylaxis, thyroid hormone, review

## Abstract

The thyroid gland requires iodine to synthesize thyroid hormones, and iodine deficiency results in the inadequate production of thyroxine and related thyroid, metabolic, developmental, and reproductive disorders. Iodine requirements are higher in infants, children, and during pregnancy and lactation than in adult men and non-pregnant women. Iodine is available in a wide range of foods and water and is susceptible to almost complete gastric and duodenal absorption as an iodide ion. A healthy diet usually provides a daily iodine consumption not exceeding 50% of the recommended intake. Iodine supplementation is usually necessary to prevent iodine deficiency disorders (IDDs), especially in endemic areas. The community-based strategy of iodine fortification in salt has eradicated IDDs, such as endemic goiter and cretinism, in countries providing adequate measures of iodine prophylaxis over several decades in the 20th century. Iodized salt is the cornerstone of iodine prophylaxis in endemic areas, and the continuous monitoring of community iodine intake and its related clinical outcomes is essential. Despite the relevant improvement in clinical outcomes, subclinical iodine deficiency persists even in Western Europe, especially among girls and women, being an issue in certain physiological conditions, such as pregnancy and lactation, and in people consuming unbalanced vegetable-based or salt-restricted diets. Detailed strategies to implement iodine intake (supplementation) could be considered for specific population groups when iodized salt alone is insufficient to provide adequate requirements.

## 1. Introduction

The thyroid gland requires iodine for the synthesis of thyroid hormones. Thyroxine (T4) is the main thyroid hormone directly synthesized by the thyroid gland. In contrast, triiodothyronine (T3), the physiologically active thyroid hormone, is produced either directly by the thyroid or after the peripheral deiodination of circulating T4 via selenium-containing deiodinases. Thyroid hormones regulate several physiologic processes, including growth, development, metabolism, and reproductive function [1].

Thyroid hormone synthesis is enhanced by the pituitary-derived thyroid-stimulating hormone (TSH) that, in turn, stimulates iodine trapping and oxidation by thyrocytes, thyroglobulin synthesis, iodothyronine coupling, and thyroid hormone release by the gland [2]. Thyroid avidity and the trapping of iodine are upregulated in iodine deficiency and suppressed in cases of overexposure.

Iodine deficiency results in the inadequate production of T4. In response to decreased blood T4 levels, the pituitary gland increases TSH to restore the circulating levels of T4. When TSH is persistently elevated, it results in thyroid enlargement (hyperplasia) and multinodular goiter [2]. As adaptation is inadequate to provide the body with sufficient thyroid hormone, iodine deficiency may result in primary hypothyroidism.

## 2. Overview of Thyroid Hormone Synthesis

Thyroid hormone synthesis requires two proteins: thyroglobulin (Tg) and thyroid peroxidase (TPO). The synthesis of both occurs under TSH control. Tg is a 660 KDa glycoprotein secreted into the follicular lumen, whose tyrosyl residues serve as a substrate for iodination and hormone synthesis. TPO is a heme-containing enzyme expressed at the apical membrane of thyrocytes. TPO reduces the H_2_O_2_ generated by NADPH oxidase to create iodinating species and catalyzes the iodination of tyrosyl residues of growing Tg molecules [2]. Oxidized iodine is incorporated into thyroxine residues to form mono- (MIT) and diiodothyronine (DIT) before they merge to generate T3 and T4. Iodothyronine is part of Tg and stored in the colloid in the follicular lumen for weeks or months, according to the individual requirement of thyroid hormones. 

The first step in thyroid hormone release is the endocytosis of colloidal droplets from the follicular lumen to the cytoplasm. Endocytic vesicles merge with lysosomes, where Tg is proteolyzed by endo- and exopeptidases. After proteolysis, thyroid hormones are released into the cytoplasm of thyrocytes, where specific carriers mediate the release of T4 into the circulation [2]. Iodine deficiency decreases the DIT-to-MIT and T4-to-T3 ratios, while iodine replacement increases them.

## 3. Iodine Metabolism and Function

Iodine is a non-metallic trace element, essential for animals and humans. Iodine accounts for around two-thirds of the molecular weight of thyroid hormones. According to the official nomenclature system, the term iodide refers to the natural form of the free element (inorganic) in its ionic state (I-), while iodine includes both inorganic iodide and iodine covalently bound to tyrosine [2].

Iodine is ingested as an inorganic ion or organically bound compound, but it is absorbed in the form of iodide after the reduction in iodine compounds in the stomach. The enteric absorption of iodide takes place in the stomach and duodenum, where the enteric isoform of the sodium iodide symporter (NIS) is largely expressed [3].

Iodine accumulates in the thyroid gland, which consequently contains the largest pool of intracellular iodine in the human body. However, the most significant amount of iodide is held in the extracellular fluid, where its concentration is around 10–15 µg/L. Circulating iodide undergoes renal clearance, while a small part is lost through the skin, intestinal secretions, or expired air. The mammary gland can also accumulate and secrete iodide, thus offering an additional source of iodine clearance in lactating women [2]. The renal clearance of iodide is 30–50 mL per minute [4] but largely depends on the individual glomerular filtration rate, without any evidence of tubular secretion or active transport [5]. Reabsorption is partial and passive, and the renal clearance of iodide is influenced by the overall iodide status [6].

The thyroid clearance of iodine is around 10–20 mL/min, but it depends on chronic iodine consumption, ranging from 3 mL/min in cases of chronic overexposure to large amounts of iodine (more than 500 μg/day), to 100 mL/min in cases of severe iodine deficiency [7]. Iodide uptake in the thyroid gland occurs through a specific carrier, the NIS. The NIS is expressed at the basolateral plasma membrane in thyrocytes. It belongs to the so-called secondary active transporter family, as the NIS uses the electrochemical gradient generated by the sodium–potassium ATPase to actively transport iodide against the gradient [8]. This mechanism is essential to maintain the intrathyroidal concentration of free iodide at 20–50 times higher than the plasma concentration [9]. The expression of the NIS is enhanced by TSH [10].

There is also an inner autoregulatory mechanism through which iodide transport and intrathyroidal metabolism inversely fluctuate with the glandular content of organic iodine. This mechanism, also known as the Wolff–Chaikoff effect, depends on the iodine saturation of the carriers and enzymes involved in iodine organification and thyroid hormone synthesis. It is an intrathyroidal defensive mechanism to protect against thyroid hormone overproduction in cases of acute or intermittent iodine overexposure [11].

Once iodide accumulates in thyrocytes, the iodine transition from the cytoplasm to the follicular lumen is facilitated by the apical iodide transporter (AIT) [12] and by pendrin [13].

Iodide is also generated via the intrathyroidal deiodination of iodothyronine after thyroglobulin hydrolyzation. Part of the circulating iodide pool undergoes re-organification into de novo synthesized iodothyronine, while the remnant spreads into the systemic circulation (iodide leakage). Iodine also originates from the peripheral degradation of thyroid hormones and enters the circulation, where it can be either recycled after subsequent thyroid uptake or finally excreted in the urine.

## 4. Natural and Artificial Sources

Iodine occurs naturally as iodide and iodate in igneous rocks and soils. However, iodine could be mobilized from superficial layers of ground, and stones such as iodide and iodate are highly soluble in the aqueous phase. Thus, they drain from rainwater into surface waters, seas, and oceans, eventually becoming available for animal and human consumption [14]. Free elemental iodine also sublimates in the atmosphere directly from soils and rocks, because of its high volatility. When rainfalls occur, iodine precipitates on the land surface and drains into the ground and rocks, and can then be assimilated by plants.

Vegetables do not provide an adequate dietary iodine supply, and vegans are exposed to iodine deficiency even in iodine-sufficient areas [15]. Meat, milk, eggs, fish, and other animal-derived foods are the most important dietary sources of iodine in human nutrition. The estimated mean concentration of iodine in animal tissues other than the thyroid (i.e., skeletal muscle) is approximately 0.1 mg/kg [16]. However, the iodine content in animal tissues depends on the iodine supplementation of background animal feed [16]. Seafood and saltwater fish are the most relevant iodine sources, as marine fauna and flora accumulate large amounts of soluble iodine from seawaters. Fresh and farmed fish, as compared to seawater foods, contain less iodine. Thus, fish from rivers or lakes usually have a lower content of this element [17,18].

Iodine intake varies according to geographical areas, but also among individuals in a specific geographic region, and, indeed, individual consumption differs daily. Iodine intake largely depends on age too [19,20,21,22]. In Germany, milk and dairy products provide around 35% of the daily requirement of iodine. The other two-thirds are supplied by meat and meat products, bread and cereals, and fish [19]. In Denmark, milk provides more than 30% of daily iodine intake [20], and a similar percentage has been reported in Swiss children [21]. In Dutch schoolchildren, seafood is a negligible source of iodine, as it is consumed only about once a month [22]. 

Thanks to alimentary policies allowing the addition of iodine to foods, processed foods containing significantly higher levels of iodine have been available in the last few decades and have been used to provide iodine prophylaxis to counteract, in nationally based programs, the clinical consequences of iodine deficiency. The iodization of salt for human food consumption is the worldwide strategy recommended for this purpose.

Iodine may enter the body through chronic consumption or exposure to certain medications, such as amiodarone, povidone–iodine, iodine-based radiocontrast media, and multivitamin preparations. For example, 200 mg of amiodarone (the mean daily dose of maintenance treatment) contains 75 mg of iodine, exceeding five-hundred-fold the recommended daily requirement of the element. Iodine-based radiocontrast media contain grams of iodine.

## 5. Recommended Intake

Daily iodine intake ranges from less than ten micrograms in extreme iodine deficiency areas to several hundred milligrams in patients taking iodine-containing medications. Generally, 150 µg of iodine is the recommended daily intake for adults and the elderly. In pregnant or lactating women, the iodine requirement increases to at least 200–250 µg daily [23]. The iodine requirement per kilogram of body weight is higher in newborns and children than in adults, corresponding to an absolute iodine intake requirement of 70–120 µg in children and 40 µg in newborns [24]. These recommendations consider the daily thyroid hormone turnover in healthy individuals, with a mean iodine intake associated with the lowest values of TSH in the normal range, the smallest thyroid volume, and the lowest incidence of transient hypothyroidism in neonatal screening, and the mean requirement of levothyroxine to restore euthyroidism in patients with thyroid agenesis or following thyroidectomy [23].

## 6. Iodine Deficiency

A healthy diet in historically iodine deficiency regions provides around 50% of the daily iodine requirement in adults, which is insufficient to ensure an adequate supply of the micronutrient. This issue is particularly relevant in certain conditions such as pregnancy and lactation when the iodine requirement is nearly double.

Several biomarkers have been used to assess daily population iodine intake. As an example, the rate of urinary iodine excretion is a reliable measure of daily iodine intake, as 90% of circulating iodine is excreted in urine [2]. The most useful laboratory markers of iodine exposure in a community-based screening program are the 24 h urinary iodine concentration and the iodine-to-creatinine urinary ratio. However, spot urinary iodine concentration assessment for population surveys is preferable to 24 h samples, as the former are impractical [25]. In iodine-sufficient regions, the median 24 h iodine concentration is equal to or more than 100 µg/L, corresponding to a daily intake of at least 130 µg.

According to the WHO, iodine deficiency disorders (IDDs), including goiter, hypothyroidism, intellective impairment, reproductive impairment, decreased child survival, and varying degrees of growth and developmental abnormalities, affect more than one billion people around the world [26].

Iodized salt has significantly reduced the prevalence of iodine deficiency in many iodine-deficient countries worldwide [23,27]. However, almost one-third of the global population still lives in geographic areas where iodine deficiency and related disorders are endemic [28].

Diffuse or nodular thyroid enlargement is the first and most common pathophysiological consequence of iodine deficiency. As mentioned above, iodine deficiency reduces the intrathyroidal synthesis of T4, with a consequent adaptive increase in serum TSH concentrations. If undiagnosed, TSH elevation for months or years is sufficient to stimulate thyroid hyperplasia and enlargement. This adaptive response is usually adequate to preserve euthyroidism over several years when subclinical iodine deficiency occurs. “Endemic” goiter refers to an epidemiological condition where more than 5% of school-aged children are diagnosed with enlarged thyroid glands in a population [29]. Moderate or severe iodine deficiency may result in primary hypothyroidism, as TSH stimulation and thyroid enlargement are insufficient to ensure euthyroidism. 

Besides iodine deficiency, other agents are defined as goitrogenic in humans and may precipitate thyroid disorders when occurring concomitantly with iodine deficiency. These agents include thiocyanate, isothiocyanates, polyphenols, phthalate esters, polychlorinated and polybrominated biphenyls, organochlorines, polycyclic aromatic hydrocarbons, and lithium [30,31,32] (Table 1). Meanwhile, thiocyanate, isothiocyanate, perchlorate, and lithium, as a few examples, inhibit iodide transport by the NIS. Phenolic compounds and phthalates hamper the oxidation and organification of iodine, and lithium affects the enzymatic proteolysis of Tg and blunts T4 release. Polybrominated biphenyls increase the rate of thyroid hormone metabolism [33]. Iodine fortification is a therapeutic strategy to prevent thyroid enlargement in patients chronically exposed to goitrogenic substances, particularly when iodine uptake and metabolism are affected (e.g., by perchlorate, lithium, and thiocyanate) [30].

Iodine deficiency in the early stages of life may significantly affect brain development. Thyroid hormones are necessary for the myelination of the central nervous system, which takes place before and shortly after birth. Primary hypothyroidism related to iodine deficiency has been found to negatively affect cognitive function, with potentially irreversible intellective consequences [34,35]. Adequate maternal exposure to iodine in the early stages of pregnancy is essential for the proper intellective development of the child, irrespective of hypothyroidism. In a longitudinal study from the UK, the verbal intelligence quotient, reading accuracy, and comprehension were significantly lower in children of women with an iodine-to-creatinine ratio of less than 150 µg/g than in women with a ratio equal to or more than 150 µg/g [36].

Iodine deficiency has also been associated with increased miscarriage and stillbirth rates, and congenital disabilities, including congenital hypothyroidism in the offspring [37,38] (Table 2). Congenital hypothyroidism comprises two classical clinical features with specific phenotypes: neurological and myxedematous. The first is characterized by intellectual impairment and developmental delays, and various neurological defects, including the underdevelopment of the cochlea leading to deafness, defects of the cerebral neocortex with intellective impairment, and the underdevelopment of the corpus striatum with motor disorders [39]. Patients do not exhibit signs of hypothyroidism, and the prevalence of goiter is similar to that observed in the general population. The hypothyroid phenotype includes dwarfism with delayed bone and sexual maturation, intellective impairment, and overt hypothyroidism. Thyroid development is critically involved, and patients usually exhibit a low thyroid volume or thyroid atrophy [40]. Neurological cretinism is related to thyroid hormone deficiency in the early stages of embryonal development, resulting from a severe maternal iodine deficiency in a phase when thyroid development is still incomplete [41]. Myxedematous cretinism is associated with thyroid insufficiency during late pregnancy or early infancy [42]. Pure forms of myxedematous cretinism predominate in Central Africa, while in other endemic regions such as New Guinea and some of South America, only neurological cretinism is described. Mixed forms were observed in India [43]. The specific geographic distribution of these different phenotypes suggests that factors other than iodine deficiency could be involved, including hereditary factors, a diet with a rich thiocyanate [44], and poor selenium, zinc, copper, manganese, iron, and antioxidant content (e.g., vitamin A) [45,46].

The prevalence of endemic goiter and other IDDs is extremely low in most European countries, while subclinical iodine deficiency remains a widespread medical issue in Western and Central Europe [47]. However, iodine deficiency is still a public health concern even in Europe. Firstly, iodine intake should be quite low in specific subgroups of the population, such as people consuming a vegan diet without the consumption of iodized salt or use of supplements containing iodine and other micronutrients (such as selenium and zinc) [48] and those with unsatisfactory adherence to dietary recommendations or with an increased iodine requirement (e.g., during pregnancy and lactation) [49]. A recent systematic review of national surveys and subnational studies confirmed that in Europe, some subjects have an iodine intake below recommended levels, especially among girls and women [50].

**Table 2:** Overview of iodine deficiency disorders by age [51].

Stage of Development	Clinical Disorders
Fetus	Congenital disabilities, high perinatal mortality, and cretinism
Newborns	Hypothyroidism, goiter, and intellective impairment
Children and adolescent	Hypothyroidism, goiter, impaired cognitive function, and delayed physical development
Adults	Hypothyroidism, goiter, iodine-induced hyperthyroidism (after iodine replacement), infertility, and subfertility
Elders	Multinodular goiter, autonomously functioning thyroid nodule, and hyperthyroidism
Pregnant women	Abortion and stillbirth

Iodine deficiency disorders (IDDs) are well-recognized clinical outcomes related to iodine deficiency. IDDs differ according to the stage of life when iodine deficiency occurs. A wide range of clinical manifestations has been described, including intellective impairment, neurological and physical defects, hypothyroidism, goiter, autonomously functioning thyroid nodule, hyperthyroidism, and reproductive complaints.

## 7. Iodine Excess

In most regions, habitual diets provide a low normal iodine supply and are more prone to induce iodine deficiency than excess [52]. Nevertheless, people living in some regions could be exposed to extraordinary iodine overload due to their diet. Chronic iodine overload is usually well tolerated, as most people exposed to a large amount of iodine do not manifest any thyroid complaints [53]. However, chronic overexposure may increase the risk of subclinical hypothyroidism and possibly goiter due to persistent TSH overstimulation [54].

Acute iodine poisoning is a rare emergency occurring after ingesting grams of iodide. Common clinical manifestations include a burning mouth, sore throat, fever, nausea, vomiting, diarrhea, and, in severe cases, coma [23]. Acutely iodide excess inhibits thyroid hormone synthesis abruptly, due to the described Wolff–Chaikoff effect. It is usually transient and reversible, but it could be persistent in certain conditions, such as chronic autoimmune thyroiditis [23].

The tolerable daily dose of iodine is around 200 µg for infants up to 3 years of age, 250 µg for those aged 4–6 years, 300 µg for 7–10 years, 450 µg for 11–14 years, 500 µg for 15–17 years, and 600 µg for adults, including pregnant or lactating women [26].

## 8. Iodine Prophylaxis

A thousand years have passed since the first medical descriptions of a relevant reduction in goiter size in patients consuming significant amounts of seaweed and sea sponges, typical products from Asian coastal regions. However, iodine was incidentally discovered in 1811 by Courtois, and it was characterized and described as a new element two years later by Gay-Lussac [55]. Jean-Francois Coindet, a Swiss physician born and practicing in Geneva, was the first to speculate that the historically described decrease in goiter size after the ingestion of seaweed was attributable to its high iodine content [56]. Hence, he created the first “therapeutic” solution of iodine by dissolving 48 grains (3.1 g) of iodine in a volumetric ounce (around 28 mL) of distilled alcohol. Based on empiric and anecdotal case series, Coindet provided the first evidence of the effectiveness of iodine fortification in reducing goiter size in goitrous patients. News of Coindet’s experience rapidly spread through Europe, prompting criticism, especially due to safety concerns in terms of overexposure to iodine. This delayed the widespread use of fortification as a basic treatment of multinodular goiter. Years later, more detailed studies were carried out by David Marine, who performed a clinical trial of an iodine prophylaxis program for schoolgirls in 1917 [56]. He found that iodine prophylaxis prevented goiter development in children with an initially normal thyroid size and induced a considerable decrease in thyroid size in around two-thirds of schoolgirls with an originally enlarged thyroid [56].

In the United States, iodine prophylaxis was started in 1924 in Michigan, which belongs to the so-named goiter belt, a group of states in which endemic goiter was highly prevalent. For the first time, fortified (iodized) salt was employed for administering iodine prophylaxis; the iodine concentration was 100 mg for each kg of salt, resulting in an estimated average intake of 500 µg iodine daily since, at that time, the mean recommended salt consumption was approximately 6.5 g per day. Iodized salt consumption has increased remarkably since the 1950s. Thereafter, the consumption of iodized salt as the main salt for household use has remained stable at around 50% [57]. The US FDA recommends fortifying iodized salt with a range of 46–76 mg iodide/kg [58].

Iodine prophylaxis programs in Europe began in the regions recognized as endemic areas for iodine deficiency since the 1920s, such as Switzerland (1922), Austria (1923, discontinued after a few years and stably reintroduced in 1963), and the Netherlands (1928). Years later, iodine prophylaxis was introduced in other countries, including Poland (1935), Finland (1940), Portugal (1971), Italy (1972), Germany (1980), and Spain (1982). Iodized salt consumption was initially voluntary, and the iodine content of fortified salt was usually insufficient to prevent or treat endemic goiter, especially in moderately endemic areas. The iodine content of fortified salt differs considerably across Europe, ranging from 10 mg iodine/kg in Austria to 60 mg iodine/kg in Spain. The difference is based on the severity of iodine deficiency, dietary policies, and information campaigns to promote iodine prophylaxis [59]. 

Iodized salt manufacturing was formally allowed by law in 1972 in Italy. Iodine prophylaxis started selectively in endemic regions after that and, five years later, was extended to the whole country. The iodine content of fortified salt was 15 mg/kg, and iodized salt consumption was on a voluntary basis. Epidemiological data in 1994 were collected and analyzed from Pescopagano, a small village of Basilicata. Analysis of about 1400 citizens living with subclinical iodine deficiency, who never underwent iodine prophylaxis, showed that iodine deficiency (mean urinary iodine excretion 55 µg/L) leads to a progressive increase in goiter prevalence with aging, a high frequency of autonomously functioning thyroid nodules and other forms of hyperthyroidism, and thyroid autoimmunity [60]. Other epidemiological reports confirmed a direct relationship between the iodine deficiency severity and the prevalence of anatomic and functional thyroid disorders and intellectual impairment [61]. The results of 10-year iodine prophylaxis in correcting iodine deficiency found that it lessened the risk of endemic goiter in schoolchildren, suggesting that the widespread use of iodized foods would be desirable to reduce IDDs [62]. At that time, a new Ministry Decree (1991) established that the iodine content of fortified salt should be increased to 30 mg/kg, but iodine fortification was still on a voluntary basis.

Law 55, promulged in late March 2005, reorganized and regulated iodine prophylaxis to reduce the risks related to iodine deficiency. Then, strict monitoring of the effect of iodine prophylaxis and information campaigns to promote iodine consumption were carried out. In 2009, the Ministry of Health instituted the National Observatory for iodine prophylaxis monitoring at the National Institute of Health to collect and analyze the effect of iodine prophylaxis over time. Salt market reports before the legislation found that iodized salt consumption was significantly lower than 50% of the salt consumed. Iodine sufficiency was found only in three Italian regions (Liguria, Tuscany, and Sicily). However, six of nine regions (Liguria, Emilia–Romagna, Marche, Tuscany, Calabria, and Sicily) were areas with endemic goiter [63,64]. In collaboration with the regional observatories, the post-law surveillance data were collected by the National Observatory for Monitoring Iodine Prophylaxis and analyzed from 2015 to 2019. Salt market reports displayed a relevant increase in iodized salt consumption (65% of the whole pool of commercialized salt). National household consumption rose to 63%, ranging from 50% (Sicily) to over 75% (Veneto and Tuscany), while the national percentage of school dining halls using iodized salt was 78%, with regional differences ranging from 65% in Sardinia to 97% in Sicily [64].

The mean urinary iodine concentration was 124 µg/L, indicating the achievement of an adequate iodine intake without any differences between rural and urban areas. A sufficient iodine intake was reached in Veneto, Emilia–Romagna, Umbria, Marche, Lazio, and Calabria, and iodine deficiency was resolved in Tuscany, Liguria, and Sicily [64,65,66,67]. In seven of the nine examined regions (Liguria, Sicily, Tuscany, Emilia–Romagna, Umbria, Marche, and Lazio), the prevalence of goiter diagnosed in schoolchildren was lower than 5%, suggesting a relevant decrease in the number of endemic areas for goiter [63]. The frequency of neonatal TSH > 5 mUI/L, an indicator of insufficient exposure to iodine during pregnancy, decreased from 6.1% in 2010 to 4.9% in 2018. Despite these improvements, the safe threshold of 3% was still far distant, suggesting a need for additional supplementation by healthcare providers, including obstetricians, gynecologists, and pediatricians [68]. 

The number of prescriptions for antithyroid medications, an indirect indicator of incident hyperthyroidism, decreased over time in Italy by 7.4% (reference years: 2001 vs. 2018). The prescription rate decreased considerably (over 10%) in seven areas, namely the Province of Trento (−16,5%), Basilicata (−13.9%), Province of Bolzano (−12.7%), Tuscany (−12.5%), Sardinia (−11.8%), Liguria (−11.3%), and Friuli Venezia Giulia (−10.5%) [61].

Since 1990, universal iodine fortification programs have provided remarkable progress worldwide, with a growing number of countries adhering to mandatory salt iodization ranging from 15 to 40 mg/kg. The number of countries that achieved adequate (median urinary iodine concentration of 100–199 μg/L) and more than adequate (median urinary iodine concentration of 200–299 μg/L) iodine intake increased remarkably in the following decades [69]. It has been estimated that 88% of the global population used iodized salt in 2018, with the highest consumption in East Asia and the Pacific areas (92%) and the lowest coverage in West and Central Africa (78%) [70]. According to the 2021 Global scorecard of iodine nutrition, in school-aged children, 146 countries reached adequate iodine exposure (defined as a median urinary iodine concentration of 100–300 μg/L), while 26 are still endemic for mild-to-moderate iodine deficiency [71].

## 9. Risk Related to Iodine Prophylaxis and Potential Iodine Overexposure

Iodine fortification programs in iodine-deficient regions recommend a daily iodine intake of 150–200 µg. Iodine fortification has been associated with an increased incidence of iodine-induced hyperthyroidism, especially in older people with a background multinodular goiter. Subclinical iodine deficiency generates a chronic stimulation leading to follicular hyperplasia, thyroid enlargement, and multinodular goiter. In natural history, one or more hyperplastic nodules may acquire an autonomous activity, thus becoming unresponsive to the normal thyroid regulation system. When iodine fortification occurs, iodine uptake and thyroid hormone synthesis are significantly enhanced, especially in the autonomous nodules of the thyroid gland, resulting in hyperthyroidism after iodine fortification [72]. The effect is brief and usually disappears after a few weeks or months, but it can result in adverse consequences in predisposed individuals (e.g., in patients at high risk of atrial fibrillation) [73].

Epidemiological data indicate that a higher incidence of autoimmune thyroid diseases is observed in people with a sufficient dietary iodine intake than in those with subclinical iodine deficiency [74]. On the other hand, chronic exposure to iodine in previously iodine-deficient patients with autoimmune thyroid disease may increase the risk of hypothyroidism and goiter, particularly in the short term [74]. It has been hypothesized that iodine exposure may trigger thyroid autoimmunity by exacerbating the immunogenicity of intrathyroidal iodized proteins, especially thyroglobulin [74]. Although some studies have demonstrated that iodine prophylaxis may increase the incidence of autoimmune thyroid diseases, other long-term trials have not confirmed that iodine prophylaxis reduces the incidence of hypothyroidism or that it does not increase the risk of hypothyroidism and thyroid autoimmunity [74].

Iodine deficiency is associated with a higher risk of follicular thyroid cancer, while iodine fortification reduces it. However, in countries previously defined as iodine-deficient regions, iodine prophylaxis has increased the prevalence of papillary thyroid cancer [73]. Moreover, a positive relationship between daily iodine intake and occult papillary thyroid cancer has also been described. Data from autopsy registries suggested that the prevalence of occult thyroid cancer was particularly relevant in Finland (36%), where iodine exposure has been substantially optimal since the 1980s [74]. According to the WHO classification, all follicular thyroid cancers presenting a papillary component were considered papillary thyroid cancers, and this contributed to an increase in the ratio of papillary to follicular thyroid in many countries after the classification change [75]. On the other hand, it should be borne in mind that most occult cancers were microcarcinoma (mostly <5 mm). This epidemiological phenomenon does not raise warnings as, first, iodine deficiency is a risk factor for follicular thyroid cancer, and second, the prognosis of papillary cancer is usually slightly better than that described for iodine-deficiency-related follicular thyroid cancer.

## 10. Conclusions

Thyroid hormones play a central role in regulating several functions in the human body, and a sufficient iodine intake is essential to maintain thyroid homeostasis. Iodine deficiency is an epidemiological issue not only in low- or middle-income countries. Even in high-income countries, where iodine fortification has gained general acceptance and diffusion, and that have experienced a significant improvement in IDD epidemiology over time, dietary habits such as a vegan diet, low consumption of iodine-rich foods, and the lack or discontinuation of measures to monitor iodine intake in a population-based manner (e.g., screening of iodine exposure) could be responsible for subclinical iodine deficiency and other IDDs.

The iodization of salt for human consumption remains the recommended strategy for adequate iodine exposure. Despite some concerns related to high iodine exposure risks (hyperthyroidism, thyroid autoimmunity, and a relative increase in the risk of papillary thyroid cancer), the benefits outweigh the risks.

Specific recommendations and strategies to implement iodine intake (as a supplement) are needed for categories of people in whom iodized salt alone appears insufficient to provide adequate requirements. 

## Figures and Tables

**Table 1:** List of foods containing goitrogenic substances.

Goitrogens	Foods
Thiocyanate	Cabbage, kale, broccoli, Brussels sprouts, cassava, soybean sprouts, turnips, and mustard
Isothiocyanates	Broccoli, watercress, brussels sprouts, cabbage, Japanese radish, and cauliflower
Polyphenols	Berries, spices, nuts, and seeds
Phthalate esters	Food packaging
Polybrominated and polychlorinated products	Oils and fats, fish and shellfish, meat, and eggs
Organochlorines	Fruits and vegetables
Polycyclic aromatic hydrocarbons	Grilled meat
Lithium	Cereals, potatoes, tomatoes, and cabbage

## Data Availability

Not applicable.
